# Multifunctional natural polysaccharide peach gum-encapsulated selenium quantum dot nanocrystals monitor and scavenge reactive oxygen species in living cells

**DOI:** 10.4103/mgr.MEDGASRES-D-26-00088

**Published:** 2026-09-12

**Authors:** Keyu Lu, Yao Chen, Zihao Liu, Shurui Zhu, Biyun Li, Yan Jin, Hongyun Xuan, Zao Dai, Huihua Yuan, Min Mao

**Affiliations:** 1 School of Life Sciences, Research Center for Intelligent Information Technology, Nantong University, Nantong, Jiangsu Province, China; 2 Department of Orthopedics, Shanghai General Hospital, Shanghai Jiao Tong University School of Medicine, Shanghai, China

**Keywords:** inflammation imaging, oxidative stress, reactive oxygen species, selenium quantum dots, theranostic nanoplatform

## Abstract

Reactive oxygen species act as essential mediators in physiological processes, yet excess reactive oxygen species cause cellular damage and inflammation. Current strategies for reactive oxygen species monitoring and intervention remain limited in clinical practice. Here, we report selenium quantum dots (SeQDs) coated with peach gum (PG@SeQDs), a fluorescent nanocomposite for simultaneous reactive oxygen species detection and scavenging. SeQDs serve as fluorescent emitters and reactive oxygen species-active centers encapsulated by peach gum as a stabilizing matrix. Transmission electron microscopy and dynamic light scattering show SeQDs ~5 nm (hydrodynamic ~47 nm) and PG@SeQDs ~170 nm (hydrodynamic ~204 nm), with a zeta potential shift from –2.1 to –7.4 mV after peach gum coating, indicating enhanced colloidal stability. PG@SeQDs exhibit excitation-dependent emission, with a maximum intensity near 260 nm. Biocompatibility assays in Schwann cells show markedly higher viability for PG@SeQDs versus SeQDs and minimal hemolysis (0.24–0.32%). Antioxidant activity by 1,1-diphenyl-2-picrylhydrazyl shows concentration-dependent scavenging (56% at 40 μg/mL after 24 hours). In RSC96 cells, PG@SeQDs most effectively suppress hydrogen peroxide-induced reactive oxygen species and demonstrate time-dependent uptake with lysosomal localization and partial mitochondrial association; uptake is enhanced under inflammatory conditions (lipopolysaccharide). Collectively, PG@SeQDs represent a promising theragnostic platform for real-time reactive oxygen species imaging and management of reactive oxygen species-related inflammatory diseases, with potential for spinal cord inflammation.

## Graphical abstract

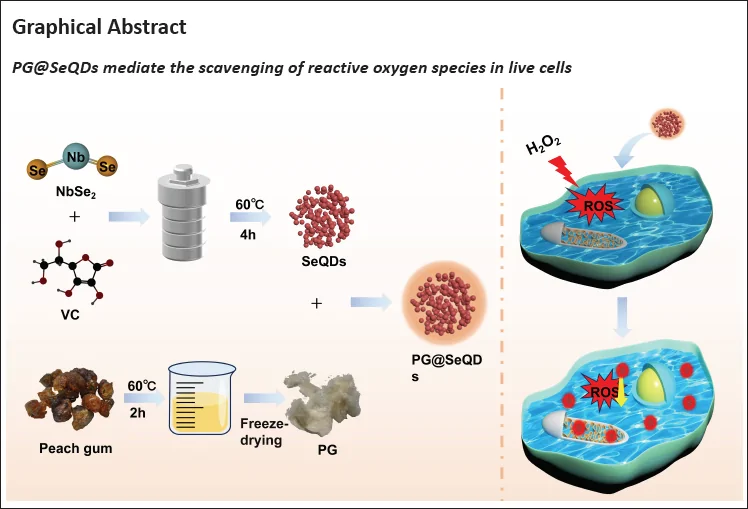

## Introduction

Reactive oxygen species (ROS) are oxygen-containing free radicals that play a crucial role in physiological oxygen metabolism. At moderate levels, ROS exert protective effects against pathogenic invaders.^1^ However, excessive ROS accumulation leads to oxidative stress,^2^ exacerbates local tissue damage,^3^ and triggers chronic inflammation.^4^,^5^,^6^ Detecting and clearing ROS in real time is essential for assessing and treating tissue inflammation.

Selenium is an essential trace element in humans that is critical for numerous physiological processes, including antioxidant defense, anticancer activity, immune enhancement, and reproductive health.^7^,^8^ It serves as a cofactor in key enzymatic complexes, such as glutathione peroxidase, thioredoxin reductase, phospholipid hydroperoxidase glutathione peroxidase, and methionine sulfoxide reductase, all of which protect cells from free radical-induced damage.^9^,^10^ Consequently, selenium plays a significant role in free radical scavenging, mitigating oxidative stress, and regulating the immune system. Selenium deficiency has been linked to various neurological disorders, including Parkinson’s disease, epilepsy, and Alzheimer’s disease,^11^,^12^ revealing its therapeutic relevance in neurology. As an integral component of selenoproteins, selenium supports central nervous system function, and its supplementation has been shown to have beneficial effects in neurological contexts. However, the benefits of selenium are dose- and form dependent, and careful dosing is required^13^ because elevated levels or certain selenium-containing formulations can exert pro-oxidant effects^14^ and cause adverse outcomes (gastrointestinal disturbances, peripheral neuropathy, dermatological effects), as documented for some selenium nanoparticles and related compounds.^15^

Nanotechnology enables the precise engineering of materials at atomic, molecular, and macromolecular scales, allowing the construction of structures with customized physical, chemical, and biological properties.^16^,^17^,^18^,^19^,^20^ Selenium nanoparticles demonstrate enhanced free radical scavenging capacity, higher bioavailability, and lower cytotoxicity relative to other selenium forms.^21^ Their ability to efficiently penetrate cells and tissues makes them particularly attractive for mitigating oxidative stress and inflammation.^22^ Further miniaturization to selenium quantum dots (SeQDs) confers additional advantages, including potent antioxidant and immunomodulatory functions and widespread cellular distribution. Moreover, the intrinsic fluorescence properties of SeQDs enable applications in real-time inflammation diagnosis, treatment, and monitoring.^23^,^24^

Composite nanoparticles capped with natural polymers offer improved hydrophilicity, biocompatibility, and low toxicity.^25^ Peach gum (PG), a natural polysaccharide exudate from peach trees, is biodegradable, environmentally friendly, and possesses notable biological activities, including antioxidant,^26^,^27^ antibacterial,^28^ hypoglycemic, and pro-proliferative effects.^29^,^30^ It also exhibits high biocompatibility and anti-inflammatory properties, with studies indicating that the PG polysaccharide DPG2 can ameliorate colitis in mice by suppressing proinflammatory cytokines such as tumor necrosis factor α, interferon γ, interleukin 1β, interleukin 6, and interleukin 17.^31^,^32^ The abundant hydroxyl and carboxyl groups in PG enable effective chelation and adsorption of metal ions, enhancing structural stability and system interactions.^33^,^34^,^35^ These characteristics make PG an ideal capping agent for synthesizing stable and functional selenium nanocrystals.

Traditional probes can monitor the dynamic changes in ROS but lack protective capabilities, while antioxidants can mitigate damage but do not allow for real-time monitoring. As a promising new type of carrier, selenium-based nanomaterials theoretically have the potential to function both as optical probes and as antioxidants. However, developing a single nanoplatform that integrates real-time ROS monitoring with efficient scavenging capabilities remains a significant challenge. To address this, we have developed a novel nanocomposite material, PG@SeQDs, aimed at combining ROS detection functionality with antioxidant activity within living cells. This study explores whether this material can simultaneously exhibit dual functions in live cells, thereby addressing the critical issue of achieving both sensing and protective functions within a single nanoreagent in this field.

## Methods

### Preparation of PG@SeQDs

SeQDs were synthesized by adding 10 mg of niobium selenide (Alfa Aesar Chemical Co., Ltd., Shanghai, China) to 100 mL of distilled water and stirring continuously for 12 hours to achieve a uniformly dispersed solution. The resulting solution was transferred into a lined autoclave with a total volume of 150 mL, which was then sealed and subjected to a constant temperature reaction at 60°C for 4 hours. After cooling naturally to room temperature, the mixture was allowed to stand for an additional 6 hours before adding 2 mL of a 40 mM vitamin C (Xilong Science Co., Ltd., Shantou, China) solution, followed by stirring for 10 minutes (pH adjusted to 8.0) and ultrasonic treatment for another 3 hours (maintaining pH at 8.0). Subsequently, the solution underwent centrifugation at 1000 × *g* for 15 minutes and was washed three times with anhydrous ethanol and water. The resultant sample was dispersed in 30 mL of ethanol, stirred thoroughly, and refluxed for an extended period of 24 hours to eliminate residual impurities. SeQDs were obtained after washing with phosphate-buffered saline (PBS; 0.01 M, pH = 7.4) three times and subsequently lyophilized.

The lyophilized SeQDs were resuspended in ethanol to obtain a SeQD suspension. For the preparation of PG@SeQDs, a mixture containing 32 mg of water-soluble PG polysaccharide (located peach orchards, Nantong, China) and 3 mg magnesium sulfate was dissolved in 5 mL of water. Then, 1 mL of SeQD suspension was added, followed by ultrasonic treatment using a cell ultrasonic crusher (model SCIENTZ-IID, Scientz Biotechnology Co., Ltd., Ningbo, China) for 3 minutes. Additionally, 0.5 mL of NaBH_4_ (0.1 M) was introduced into the mixture, which was stirred at room temperature for 10 minutes. The precipitate formed during this process was collected through acetone (Shanghai Runjie Chemical Co., Ltd., Shanghai, China) precipitation and subsequently redispersed into deionized water.

### Basic characterization of SeQDs and PG@SeQDs

The particle size and potential of SeQDs and PG@SeQDs were measured by a Zetasizer Nano particle characterization system (ZS90, Malvern Panalytical, Great Malvern, UK), and morphological characterization was performed using a transmission electron microscope (JEM-2100, JEOL Ltd., Akishima, Japan).

Ultraviolet-visible absorption spectroscopy was performed using a UV-2600 spectrophotometer (Shimadzu, Kyoto, Japan) to characterize the optical absorption properties of both SeQDs and PG@SeQDs. Spectra were recorded in the range of 200–600 nm.

Fluorescence properties were measured using an RF-5301PC fluorescence spectrophotometer. A gradient excitation wavelength (250–350 nm, interval 10 nm) was used to detect and analyze PG@SeQDs.

Fourier transform infrared spectroscopy was used to analyze the composition and surface functional groups of PG, SeQDs, and PG@SeQDs. After thorough drying, the samples were separately mixed with potassium bromide (KBr) crystal powder and ground. The four samples were processed using the tablet compression method. Infrared spectroscopic analysis was performed on the samples within the wavenumber range of 500 cm^−1^ to 4000 cm^−1^.

### Cell viability test

PG@SeQDs were placed in a 15 mL centrifuge tube, sterilized by immersion in 75% ethanol, rinsed fully with sterile PBS, and subsequently subjected to ultraviolet irradiation for 30 minutes. RSC96 cells (Wuhan Servicebio Technology Co., Ltd., Wuhan, China) were cultured in Dulbecco’s modified Eagle medium low-glucose medium containing 10% fetal bovine serum, and 1% double antibody was added to the well plate at a density of 1 × 10^4^ cells/mL. After 1 day of cultivation, 40 μg/mL PG@SeQDs was added to each well. The cells were cultured in a cell incubator and stained with a live/dead cell staining kit (Shanghai Biyuntian Biotechnology Co., Ltd., Shanghai, China) at 1, 3, and 5 days and then observed with an inverted optical microscope (ECLIPSE Ti2, Nikon Corporation, Tokyo, Japan). All cell viability assays included a control group (cell cultures without any nanoparticles) as the negative control.

### Cytocompatibility test

Schwann cells (Cat# STCC30007P, Wuhan Servicebio Technology Co., Ltd.; 1 × 10^4^ cells/mL) were seeded in a 24-well plate with PG@SeQDs after 1 day of culture at a concentration of 40 μg/mL. The cell numbers at days 1, 3, and 5 were detected by the cell counting kit-8 kit (Shanghai Biyuntian Biotechnology Co., Ltd.) to evaluate cell proliferation.

### Hemolysis test

Female Sprague–Dawley rats (6 weeks old, *n* = 6) were provided by the Laboratory Animal Center of Nantong University (Nantong, China; license No. SCXK(Su)2024-0015). All animals were housed under standard laboratory conditions (temperature 22 ± 2°C, humidity 50 ± 10%, 12-hour light/dark cycle) with free access to standard rodent chow and drinking water. Prior to blood collection, the rats were anesthetized by intraperitoneal injection of Avertin (2,2,2-tribromoethanol dissolved in tert-amyl alcohol). Blood was collected via retro-orbital sinus puncture (eyeball blood collection). Following blood collection, all rats were euthanized by cervical dislocation. All animal experiments were approved by the Laboratory Animal Ethics Committee of Nantong University (ethical approval No. P20260303-037) and performed in accordance with *the Guide for the Care and Use of Laboratory Animals* (8^th^ ed).^36^

The hemolysis test was modified from a previous method.^37^ After eyeball blood collection in rats, anticoagulant (3% (w/v) sodium citrate, 0.1% (w/v) citric acid, and 2.5% (w/v) glucose) was added to whole blood at a ratio of 7:1. Then, 2 mL of whole blood was diluted to 4.5 mL with deionized water and 0.9% saline as positive and negative controls, respectively. PG@SeQDs at various concentrations (10, 20, 40, and 80 μg/mL) and SeQDs (40 μg/mL) were placed in 10 mL 0.9% normal saline with 0.2 mL blood and incubated at 37°C for 1 hour. Then, the mixed solution was centrifuged at 90 × *g* for 5 minutes, and the absorbance at 545 nm was measured using a spectrophotometer. The hemolysis rate was calculated by (absorbance_experimental group_ – absorbance_negative group_)/(absorbance_positive group_ – absorbance_negative group_) ×100%.

### Detection of reactive oxygen species clearance *in vitro*

The 1,1-diphenyl-2-picrylhydrazyl (DPPH) method is a common method to determine the oxidation resistance of materials. The DPPH free radical scavenging ability of SeQDs or PG@SeQDs was determined according to previously reported methods.^38^ DPPH stock solution was prepared by dissolving 3.5 mg of DPPH in anhydrous ethanol and quantitatively transferring it to a 10-mL volumetric flask. A 2-mL aliquot of the stock solution was then diluted to 100 mL with anhydrous ethanol to obtain a working solution of 0.0178 mM DPPH. PG@SeQDs at various concentrations (0, 20, 40, 100, and 200 μg/mL) were immersed in 10 mL of the DPPH working solution for 1 hour. The absorbance of the supernatant was measured at 517 nm at 3, 6, 12, and 24 hours. The antioxidant activity was calculated by the following formula: DPPH free radical scavenging rate (%) = (*A*_0_ – *A*_1_)/*A*_0_ × 100, where *A*_0_ is the absorbance of DPPH/ethanol and *A*_1_ is the absorbance of the sample and DPPH/ethanol mixture.

### Intracellular reactive oxygen species detection

The accumulation of ROS in cells was monitored using the fluorescent probe 2′,7′-dichlorodihydrofluorescein diacetate (DCFH-DA). RSC96 cells were pretreated with SeQDs and PG@SeQDs (20, 40, 100, and 200 μg/mL) for 24 hours and then stained with DCFH-DA dye (Beyotime Biotechnology Co., Ltd., Shanghai, China) in darkness at 37°C for 2 hours. Finally, the cells were stimulated with 400 μL of hydrogen peroxide for 20 minutes and washed with PBS. The cells were observed under a fluorescence microscope (ECLIPSE Ti2, Nikon Corporation), excited at 488 nm and emitted at 530 nm.

### Subcellular colocalization

RSC96 cells on the slide were treated with 40 μg/mL PG@SeQDs for 24 hours and then washed twice with PBS. The mitochondria or lysosomes of the cells were stained with a relevant fluorescent probe (MitoTracker™ Green FM or LysoTracker™ Green DND-26, Shanghai Biyuntian Biotechnology Co., Ltd.) according to the manufacturer’s instructions. Then, the cells were fixed with 4% paraformaldehyde for 20 minutes and stained with 4′,6-diamidino-2-phenylindole (Beyotime Biotechnology Co., Ltd.) at room temperature for 30 minutes. The cells were observed by confocal laser scanning fluorescence microscopy (Zeiss Axio Scope A1, Carl Zeiss AG, Oberkochen, Germany).

### Lipopolysaccharide-induced inflammatory cell model

To investigate the cellular uptake behavior of PG@SeQDs under inflammatory conditions, RSC96 cells were seeded in confocal dishes at a density of 1 × 10^4^ cells/mL and cultured for 24 hours. The cells were then pretreated with lipopolysaccharide (LPS; Beyotime Biotechnology Co., Ltd.) at concentrations of 1 and 10 μg/mL for 24 hours to simulate mild and severe inflammatory microenvironments, respectively. After LPS pretreatment, the medium was removed, the cells were washed with PBS, and fresh medium containing 40 μg/mL PG@SeQDs was added. Following incubation for 24 hours, the cells were washed with PBS and observed under a laser scanning confocal microscope to evaluate intracellular fluorescence intensity.

### Statistical analysis

All quantitative data are shown as the mean ± standard deviation (SD) with at least three independent repetitions in each group (*n* ≥ 3). Student’s *t*-test or one-way analysis of variance was used to statistically analyze the data with Origin 2025 software (OriginLab, Northampton, MA, USA). A *P* value less than 0.05 was considered statistically significant.

## Results

### Preparation and characterization of SeQDs and PG@SeQDs

Quantum dots are nanoparticles ranging from 2 to 20 nm in diameter,^39^ known for their excellent biocompatibility, small size, and tunable fluorescence properties.^40^,^41^ In comparison with organic materials, inorganic nanomaterials offer ease of fabrication and retain the intrinsic properties of their constituent elements.^42^ To develop a dual-functional nanoplatform for ROS detection and scavenging, SeQDs were synthesized and subsequently capped with PG polysaccharide to form PG@SeQD nanocomposites.

SeQDs were synthesized using vitamin C as a reducing agent and high-temperature-treated niobium selenide as a template. PG@SeQDs were then prepared by encapsulating SeQDs with water-soluble PG polysaccharide as a capping agent. Transmission electron microscopy images showed that both SeQDs and PG@SeQDs exhibited uniform spherical morphologies and good dispersity (**Figure 1A** and **B**). The average particle size of the SeQDs was approximately 5 nm. The average particle size of PG@SeQDs was approximately 170 nm.

**Figure 1:**
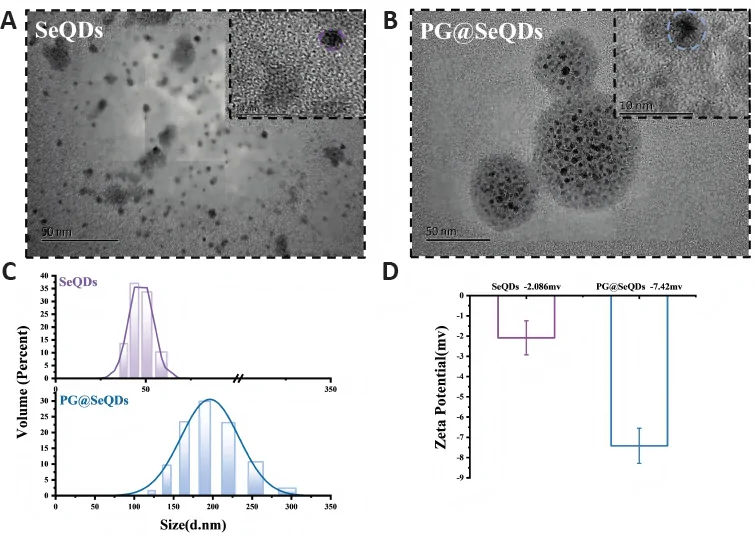
Characterization of SeQDs and PG@SeQDs. (A, B) TEM images of SeQDs (A) and PG@SeQDs (B). Scale bars: 50 nm, 10 nm (enlarged images). (C) Size of SeQDs and PG@SeQDs. (D) Zeta potential of SeQDs and PG@SeQDs. PG@SeQDs: SeQDs coated with peach gum; SeQDs: selenium quantum dots.

The size distribution and zeta potential of the two materials were analyzed using a nanoparticle size analyzer (**Figure 1C** and **D**). SeQDs had a hydrodynamic size of 47.08 nm with a zeta potential of –2.086 mV. PG@SeQDs had a hydrodynamic size of 204.5 nm with a zeta potential of –7.42 mV. The size of PG@SeQDs was consistent with the TEM results. These results confirm the successful preparation of PG@SeQDs with distinct physicochemical properties compared to bare SeQDs.

### Optical properties of PG@SeQDs

Quantum dots are well known for their distinctive quantum effects, which confer tunable emission wavelengths along with narrow and symmetrical fluorescence emission profiles.^43^ The optical properties of PG@SeQDs were systematically evaluated to determine their suitability for fluorescence-based ROS detection. The ultraviolet-visible absorption spectra of both SeQDs and PG@SeQDs are shown in **Figure 2A**. Fluorescence spectrophotometry was used to acquire the excitation and emission spectra of PG@SeQDs. A series of excitation wavelengths from 250 to 350 nm (in 10 nm increments) was applied. The fluorescence emission of PG@SeQDs exhibited a clear dependence on the excitation wavelength (**Figure 2B**). The maximum fluorescence intensity was achieved under excitation at 260 nm. This was identified as the optimal excitation wavelength. Fourier transform infrared spectroscopy was used to analyze the chemical structures of PG, SeQDs, and PG@SeQDs (**Figure 2C**). Characteristic absorption peaks were observed at 3430 cm^−1^, 1640 cm^−1^, 1079 cm^−1^, and 755 cm^−1^. These peaks correspond to O-H stretching vibration, C=O stretching, C-O stretching, and C-H vibration, respectively. The O-H band at approximately 3430 cm^−1^ broadened slightly in the composite spectrum. The C=O band remained unchanged. No new covalent peaks appeared. The zeta potential changed from –2.086 mV (bare SeQDs) to –7.42 mV (PG@SeQDs).

**Figure 2:**
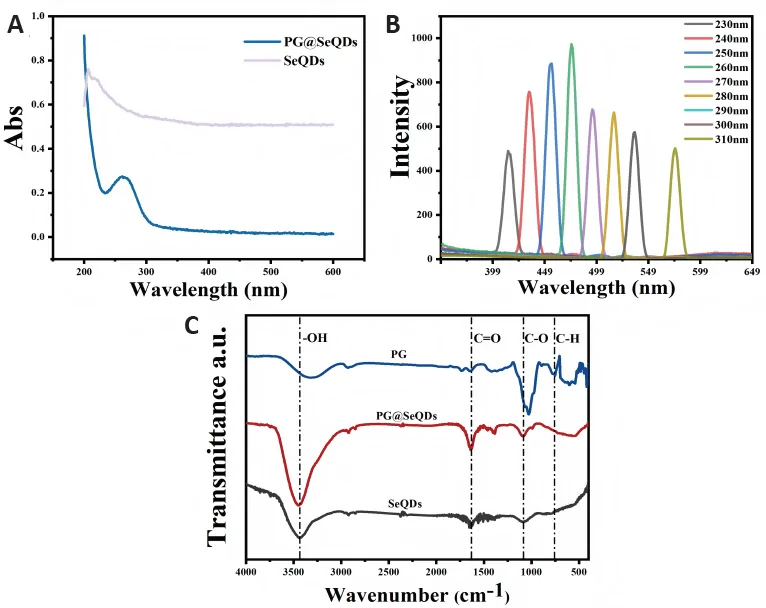
Optical properties of PG@SeQD nanocomposites. (A) Ultraviolet-visible spectra of SeQDs and PG@SeQDs. (B) Fluorescence spectrum of PG@SeQDs. (C) Fourier transform infrared spectra of PG, SeQDs and PG@SeQDs. Abs: Absorbance; PG: peach gum; PG@SeQDs: SeQDs coated with PG; SeQDs: selenium quantum dots.

### Biocompatibility and safety of PG@SeQDs

The biocompatibility and hemocompatibility of PG@SeQDs were evaluated for biomedical applications. Live/dead staining of Schwann cells cultured for 1, 3, and 5 days (**Figure 3A**) showed that while SeQDs alone exhibited limited cytocompatibility, PG@SeQDs significantly improved cell viability, as evidenced by a greater number of live cells. Quantitative cell counting kit-8 assays corroborated these findings, with PG@SeQDs inducing markedly higher absorbance at 450 nm after 5 days than SeQDs (**Figure 3B**). Hemocompatibility assessment revealed that, unlike the complete hemolysis observed with distilled water (positive control), all tested concentrations of PG@SeQDs in 0.9% normal saline remained clear, comparable to the negative control, with no obvious hemolysis (**Figure 3C**). Quantitative hemolysis rates for PG@SeQDs ranged from 0.24% to 0.32%, well below the 5.0% threshold for blood-contact materials.^44^ Collectively, these results demonstrate that PG coating significantly enhances the biocompatibility of SeQDs, conferring excellent cytocompatibility and hemocompatibility that support their potential for in vivo applications.

**Figure 3:**
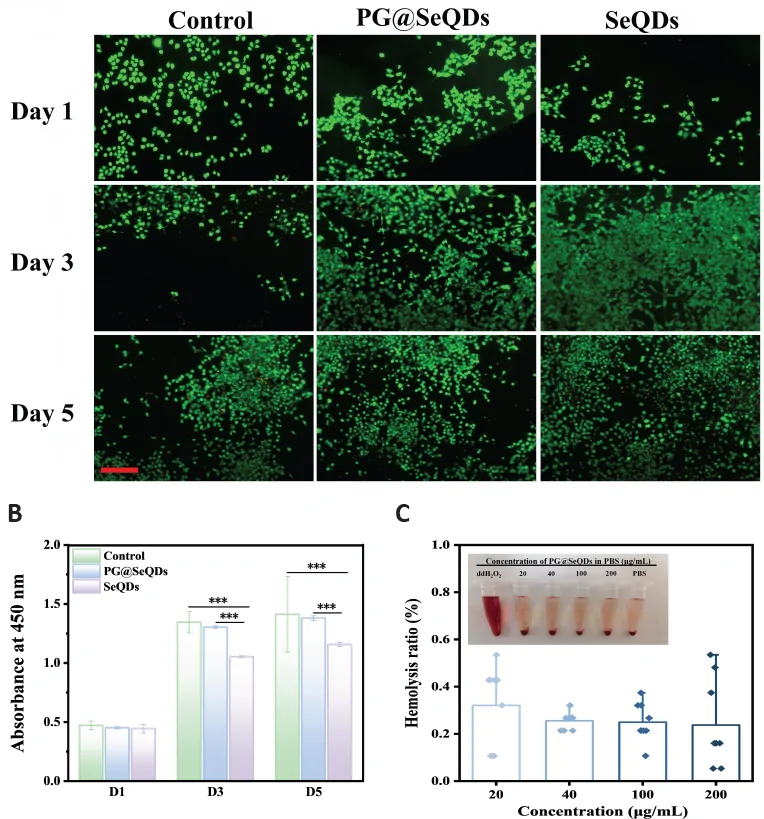
Biocompatibility of PG@SeQDs. (A) Live/dead staining of RSC96 cells. Surface functionalization with PG substantially improved the biocompatibility of SeQDs. Scale bar: 100 μm. (B) Cell Counting Kit-8 test. (C) Blood biocompatibility. Data are expressed as the mean ± SD, with three independent experiments. ***P < 0.001 (one-way analysis of variance). PG: Peach gum; PG@SeQDs: SeQDs coated with PG; SeQDs: selenium quantum dots.

### Antioxidant capacity of PG@SeQDs

The antioxidant properties of PG@SeQDs were evaluated both in cell-free systems and at the cellular level to assess their ROS scavenging capability. The DPPH free radical scavenging assay (**Figure 4**) demonstrated that PG@SeQDs effectively scavenged DPPH radicals in a concentration-dependent manner, with the sample at 40 μg/mL exhibiting the highest ROS clearance efficiency. The DPPH radical scavenging rate increased progressively over time, reaching 56% after 24 hours, and the antioxidant activity was rapidly activated within the first 4 hours. The cytoprotective effect against ROS was further validated using the fluorescent probe DCFH-DA in RSC96 cells (**Figure 5**). Cells treated with hydrogen peroxide alone or cotreated with PG exhibited strong fluorescence, confirming substantial ROS accumulation, whereas cells incubated with SeQDs or PG@SeQDs showed markedly reduced fluorescence. Notably, the PG@SeQDs group displayed the most significant ROS scavenging effect, comparable to the negative control. Collectively, these findings confirm that PG@SeQDs possess robust antioxidant properties in both cell-free and cellular environments, effectively scavenging ROS and protecting cells from oxidative damage.

**Figure 4:**
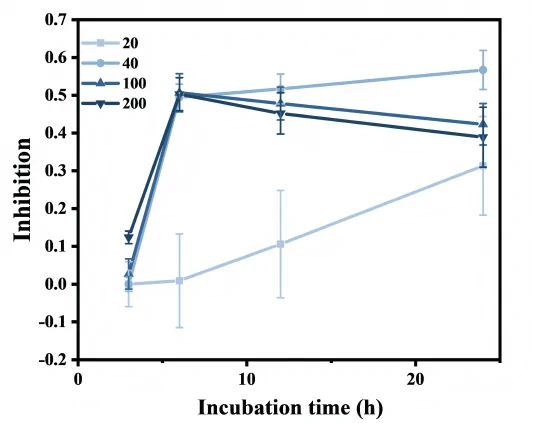
Antioxidant tests of different concentrations of PG@SeQDs. PG@SeQDs: Selenium quantum dots coated with peach gum.

**Figure 5:**
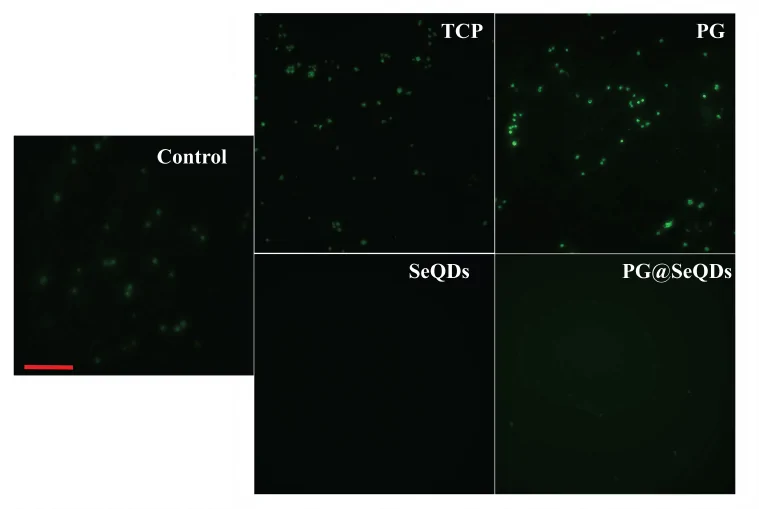
Intracellular ROS assay of PG@SeQDs. PG coating contributes to improved antioxidant performance beyond bare SeQDs. Scale bar: 100 μm. PG: Peach gum; PG@SeQDs: SeQDs coated with PG; ROS: reactive oxygen species; SeQDs: selenium quantum dots; TCP: hydrogen peroxide-treated cells.

### Endocytosis and subcellular colocalization of PG@SeQDs

The cellular internalization behavior of PG@SeQDs was investigated by leveraging their intrinsic fluorescence properties. Confocal microscopy analysis of RSC96 cells incubated with PG@SeQDs (40 μg/mL) revealed time-dependent internalization, with the nanocomposites remaining intracellularly stable for up to 24 hours, and no significant excretion was observed. Colocalization studies using organelle-specific fluorescent markers (LysoTracker for lysosomes and MitoTracker for mitochondria) showed that PG@SeQDs predominantly accumulated in lysosomes, with partial colocalization also observed in mitochondria (**Figure 6**). Furthermore, cellular uptake was evaluated in an LPS-pretreated inflammatory cell model using 1 and 10 μg/mL LPS to simulate mild and severe inflammatory microenvironments, respectively. Compared with the unstimulated control group, the 1 μg/mL LPS pretreatment group exhibited increased intracellular fluorescence intensity, and the 10 μg/mL LPS pretreatment group showed an even more significant increase. Collectively, these results demonstrate that PG@SeQDs are effectively internalized by living cells, exhibit prolonged intracellular retention, and localize within key organelles involved in ROS generation and degradation, with inflammation-enhanced uptake further supporting their potential for targeted therapeutic applications.

**Figure 6:**
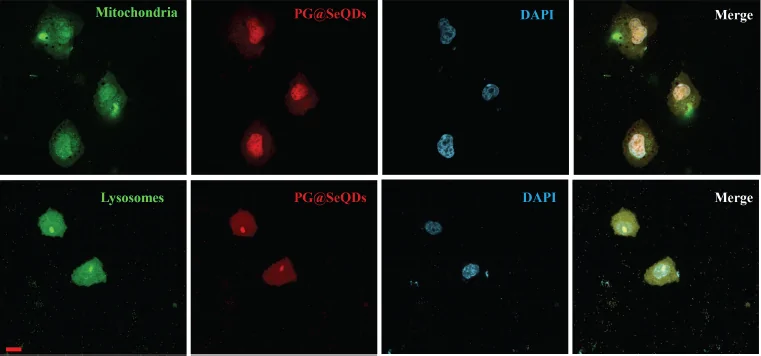
The subcellular colocalization of PG@SeQDs in RSC96 cells. PG@SeQDs were mainly found in lysosomes, although some were also observed in mitochondria. Scale bar: 10 μm. DAPI: 4’,6-Diamidino-2-phenylindole; PG@SeQDs: selenium quantum dots coated with peach gum

**Figure 7:**
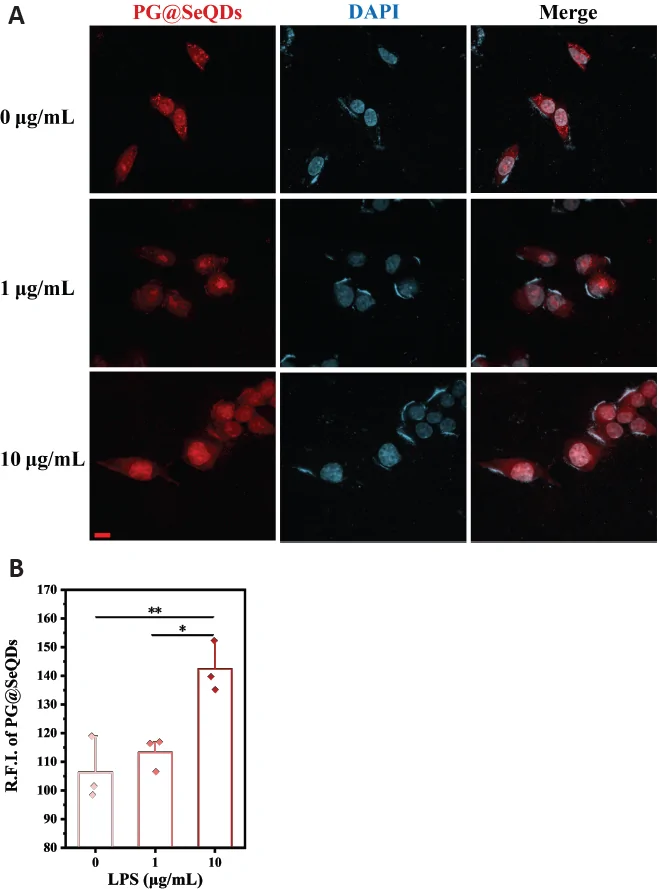
Inflammation-enhanced cellular uptake capability of PG@SeQDs. (A) Fluorescence images of cells treated with LPS at different concentrations. PG@SeQDs possess an inflammation-enhanced cellular uptake capability. Scale bar: 10 μm. (B) Fluorescence quantitative analysis of PG@SeQDs. Data are expressed as the mean ± SD, with three independent experiments. *P < 0.05, **P < 0.01 (one-way analysis of variance). DAPI: 4’,6-Diamidino-2-phenylindole; LPS: lipopolysaccharide; PG@SeQDs: selenium quantum dots coated with peach gum; R.F.I.: relative fluorescence intensity.

## Discussion

In this study, we successfully synthesized a novel nanocomposite consisting of PG@SeQDs, aiming to develop a biocompatible, antioxidant nanomaterial with favorable optical properties and cellular internalization capability. Transmission electron microscopy revealed uniform spherical morphologies for both SeQDs and PG@SeQDs, with average particle sizes of approximately 5 and 170 nm, respectively, and dynamic light scattering showed hydrodynamic diameters of 47.08 nm for SeQDs and 204.5 nm for PG@SeQDs, the latter being consistent with the transmission electron microscopy results. Notably, bare SeQDs exhibited a tendency toward aggregation in aqueous suspension, as supported by a low absolute zeta potential of –2.086 mV. After PG coating, the zeta potential decreased to –7.42 mV, approaching that of pure PG.^45^ Fourier transform infrared spectroscopy further confirmed the successful integration of PG with SeQDs, with a broadened O–H band suggesting hydrogen bonding and the absence of new covalent peaks indicating noncovalent interactions.^46^ Cytocompatibility assessment is a fundamental prerequisite for biomaterials intended for *in vivo* use.^47^ Live/dead staining and cell counting kit-8 assays consistently demonstrated that PG@SeQDs significantly promoted Schwann cell viability and proliferation compared with bare SeQDs, which exhibited limited cytocompatibility. More importantly, hemocompatibility evaluation revealed that all tested concentrations of PG@SeQDs induced hemolysis rates of only 0.24–0.32%, which are well below the 5.0% threshold for blood-contacting materials, indicating excellent erythrocyte compatibility and suggesting their potential suitability for intravenous administration or other in vivo applications. Regarding antioxidant capacity, PG@SeQDs scavenged DPPH free radicals in a concentration- and time-dependent manner, achieving 56% clearance after 24 hours, and the DCFH-DA cellular assay showed that PG@SeQDs markedly reduced hydrogen peroxide-induced ROS accumulation in RSC96 cells, with an effect superior to that of bare SeQDs and comparable to the negative control, confirming robust ROS scavenging capability. Leveraging the intrinsic fluorescence of PG@SeQDs, confocal microscopy demonstrated that cellular uptake increased progressively over time, and internalized quantum dots remained stable within cells for up to 24 hours without significant excretion. Subcellular colocalization studies indicate that PG@SeQDs primarily accumulate in lysosomes, which is consistent with the characteristics of the endocytic pathway. Additionally, a portion of PG@SeQDs colocalizes with mitochondria, the main site of superoxide anion radical production.^48^ This suggests that PG@SeQDs can selectively target key organelles involved in the generation and degradation of ROS. Furthermore, in LPS-pretreated inflammatory cell models, PG@SeQDs exhibited enhanced cellular accumulation in a concentration-dependent manner (1 and 10 μg/mL LPS), which can be attributed to increased membrane permeability and upregulated endocytic activity under inflammatory conditions, revealing the potential of PG@SeQDs for inflammation-targeted applications, particularly in severe inflammatory microenvironments such as spinal cord injury. Despite these promising results, some limitations should be acknowledged, including the need to elucidate the exact molecular mechanism of mitochondrial ROS scavenging, to systematically evaluate long-term in vivo biosafety and biodegradation and to validate the inflammation-enhanced uptake in animal models. Future work will focus on *in vivo* biodistribution and therapeutic efficacy in oxidative stress-driven diseases, as well as engineering targeting ligands to further enhance specificity for inflamed tissues.

In summary, we have developed a biocompatible, hemocompatible, and highly antioxidant PG@SeQD nanocomposite that exhibits time-dependent endocytosis, lysosomal and mitochondrial colocalization, and inflammation-enhanced cellular uptake, collectively positioning PG@SeQDs as a promising nanoplatform for real-time imaging and targeted ROS scavenging in inflammatory diseases.

## Data Availability

*All data involved with this study are available from the authors when reasonably requested.*
